# Crystal growth and optical characteristics of beryllium-free polyphosphate, KLa(PO_3_)_4_, a possible deep-ultraviolet nonlinear optical crystal

**DOI:** 10.1038/srep25201

**Published:** 2016-04-29

**Authors:** Pai Shan, Tongqing Sun, Hong Chen, Hongde Liu, Shaolin Chen, Xuanwen Liu, Yongfa Kong, Jingjun Xu

**Affiliations:** 1 The MOE Key Laboratory of Weak-Light Nonlinear Photonics and School of Physics, Nankai University, Tianjin 300071, China; 2 Collaborative Innovation Center of Extreme Optics, Shanxi University, Taiyuan 030006, China; 3 Teda Institute of Applied Physics, Nankai University, Tianjin 300457, China; 4 School of Resources and Materials, Northeastern University at Qinhuangdao, Qinhuangdao 066004, China

## Abstract

Deep-ultraviolet nonlinear optical crystals are of great importance as key materials in generating coherent light with wavelength below 200 nm through cascaded frequency conversion of solid-state lasers. However, the solely usable crystal in practice, KBe_2_BO_3_F_2_ (KBBF), is still commercially unavailable because of the high toxicity of beryllium-containing and the extreme difficulty of crystal growth. Here, we report the crystal growth and characteristics of an beryllium-free polyphosphate, KLa(PO_3_)_4_. Centimeter-sized single crystals have been easily obtained by the flux method and slow-cooling technique. The second-harmonic generation efficiency of KLa(PO_3_)_4_ powder is 0.7 times that of KH_2_PO_4_; moreover, the KLa(PO_3_)_4_ crystal is phase-matchable. Remarkably, the KLa(PO_3_)_4_ crystal exhibits an absorption edge of 162 nm, which is the shortest among phase-matchable phosphates so far. These attributes make KLa(PO_3_)_4_ a possible deep-ultraviolet nonlinear optical crystal. An analysis of the dipole moments of the polyhedra and theoretical calculations by density functional theory were made to elucidate the structure-properties relationships of KLa(PO_3_)_4_.

Coherent deep-ultraviolet (deep-UV) light with wavelength below 200 nm have gained worldwide intense concerns due to their important academic and technological applications in photochemistry, precise micro-manufacturing, semiconductor photolithography, modern scientific instruments, etc.^1^,^2^,^3^,^4^. Nonlinear optical (NLO) crystals are the key materials in producing deep-UV coherent light with solid-state lasers through cascaded frequency conversion. To be optically applicable, a deep-UV NLO crystal should have an acentric crystallographic structure, a good second-harmonic generation (SHG) response, phase-matchable capability, and basically, a deep-UV absorption edge as short as possible (i.e., a wider band gap than 6.0 eV).

To date, deep-UV NLO materials are almost exclusively limited to borates. (KBe_2_BO_3_F_2_) KBBF is still the solely practicable deep-UV material that produces coherent deep-UV light by the direct SHG process^5^. However, growing thick KBBF crystal is extremely difficult owing to its strong growth habit of layering, and thus, the coherent light output power is severely limited^6^. In addition, the component of beryllium is very poisonous. To keep wide energy gap while overcoming the structural weakness of KBBF, some noncentrosymmetric beryllium borate crystals have been found, e.g., RbBe_2_(BO_3_)F_2_, Na_2_CsBe_6_B_5_O_15_, NaSr_3_Be_3_B_3_O_9_F_4_, NaCaBe_2_B_2_O_6_F, and LiNa_5_Be_12_B_12_O_33_, which exhibit short cutoff wavelengths up to the deep-UV region^7^,^8^,^9^,^10^,^11^. However, the toxicity of beryllium is yet an obstacle to their practical applications. Other newly developed beryllium-free borates, such as, Ba_4_B_11_O_20_F, Ba_3_B_6_O_11_F_2_, Li_4_Sr(BO_3_)_2_, and Rb_3_Al_3_B_3_O_10_F, are still at the laboratory stage^12^,^13^,^14^,^15^. Furthermore, for some borates, the basic optical properties, such as UV absorption edge, are even from synthesized powders due to the difficulty of obtaining crystals with a large size and high optical quality. Therefore, there is a quite urgent demand to discover new and beryllium-free deep-UV NLO materials now.

Recently, several acentric compounds, including Na_3_Lu(CO_3_)_2_F_2_, Ba_3_P_3_O_10_Cl, RbBa_2_(PO_3_)_5_, Ba_5_P_6_O_10_, Sr_2_(OH)_3_NO_3_, and RbMgCO_3_F, have been discovered, which have rather wide energy gaps^16^,^17^,^18^,^19^,^20^,^21^. These discoveries suggest that deep-UV NLO compound should not be confined to borates. The aforementioned nitrate and carbonate fluorides present strong powder SHG responses due to the π-conjugated structural units of [CO_3_]^2−^ and [NO_3_]^−^, whereas they are easily decomposed. By contrast, the phosphates have good thermal stabilities, and can be easily grown so as to meet the requirements of experimental studies and potential applications. Thus, we pay more interest in phosphate—a novel promising candidate.

It is well-known that both rare-earth La^3+^ and alkali ions have deep-UV transparency because of their empty *f* and *d* orbits. In light of this strategy, we have studied a condensed metaphosphate of cesium and lanthanum crystal, CsLa(PO_3_)_4_, which indeed exhibits a very short absorption edge (167 nm) and moderate powder SHG efficiency^22^. Unfortunately, it is not phase-matchable. KLa(PO_3_)_4_ also belongs to the family of alkali and lanthanide polyphosphates, and has the same stoichiometric formula with CsLa(PO_3_)_4_. Though both of them just crystallize in acentric *P*2_1_ space group, their crystallographic structures are apparently distinguishing as a result of the sizes of the alkali ions. The P–O chains of CsLa(PO_3_)_4_ are helical and run along the symmetrical *b* axis^23^, while the chains of KLa(PO_3_)_4_ are zigzag and are along the *c* axis^24^. According to the structural classification for this kind of polyphosphates proposed by Palkina *et al.*^25^, CsLa(PO_3_)_4_ and KLa(PO_3_)_4_ belong to type VI and III, respectively. Hence, the optical properties of the KLa(PO_3_)_4_ crystal is more likely to differ from CsLa(PO_3_)_4_, and are worth being investigated.

Here we report the successful growth of bulk KLa(PO_3_)_4_ crystal and its properties. Its SHG response not only is stronger than that of CsLa(PO_3_)_4_, but also is phase-matchable. Moreover, it presents a remarkably short absorption edge of 162 nm, which is the shortest among the reported phase-matchable phosphates to date.

## Results and Discussion

### Crystal Growth and Morphology

**Figure 1:**
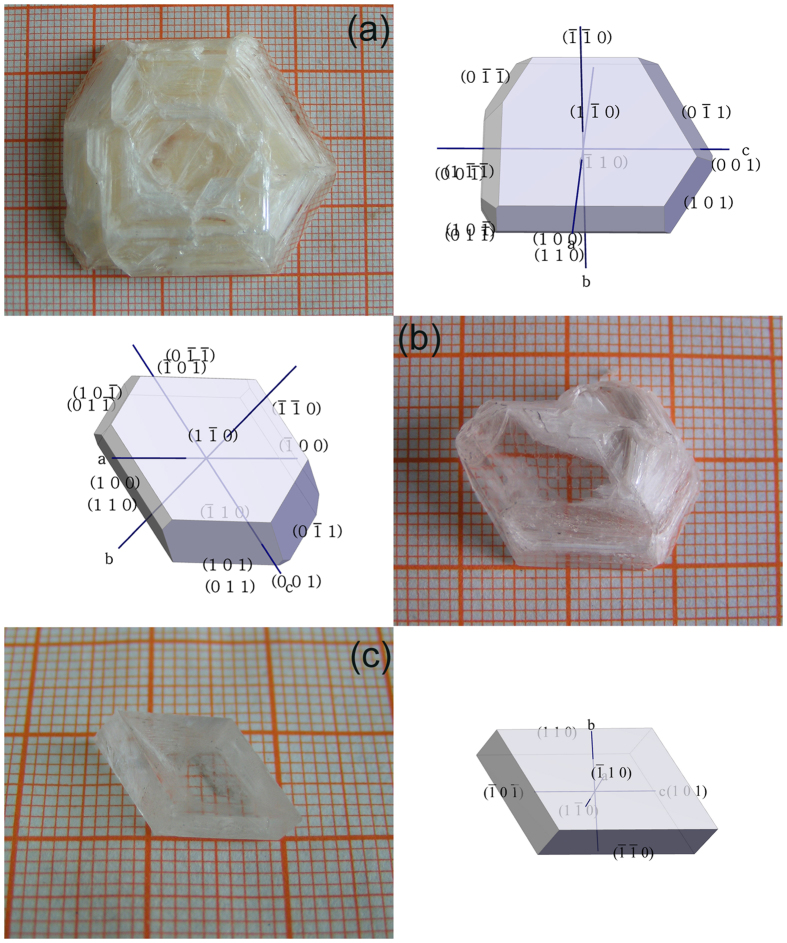
The photographs and their morphological schemes of as-grown KLa(PO_3_)_4_ crystals by different cooling rates: (a) 1 °C/1.5 days, (b) 1 °C/2 days, and (c) 1 °C/3 days. Their mass and sizes are 18.47 g and 40 × 35 × 8 mm^3^ for (**a**), 4.01 g and 19 × 21 × 7 mm^3^ for (**b**), and 0.73 g and 10 × 16 × 3 mm^3^ for (**c**), respectively.

We successfully obtained bulk crystals of KLa(PO_3_)_4_ by the flux method and slow-cooling technique. Figure 1 presents the photographs of the as-grown crystals and their associated growth data. The crystals were analyzed by powder X-ray diffraction (PXRD) and Raman spectroscopy for determining their phase compositions and structural features. The experimental PXRD pattern of the pulverized KLa(PO_3_)_4_ crystal shown in supplementary information is consistent with its standard pattern (PDF Card No. 01-075-2478), which means the as-grown crystals crystallize in the acentric *P*2_1_ space group.

One of the as-grown crystals was chosen for elemental analysis by energy dispersive X-ray spectroscope (EDS). The results confirmed that the crystal contains K, La, and P elements, and that the molar ratio of K:La:P is 1:0.94:4.27, which is consistent with the formula of KLa(PO_3_)_4_. We polished the as-grown crystal presented in Figure 1(c), which had the best crystal quality among all the crystals, and measured its density by the buoyancy method. The experimental value of density is 3.20 g·cm^−3^, which is very close to the theoretical value (3.23 g·cm^−3^). The crystals are nonhygroscopic, and can be kept in air for weeks without any changes.

Tetra-metaphosphate of potassium and lanthanum is a polymorphic compound, and there are two crystallographic structures. One is poly-metaphosphate with *P*2_1_ space group, i.e., the crystal studied here, the phosphoric anions of which have long-chain geometry of [PO_3_]_∞_, and its formula is usually written as KLa(PO_3_)_4_. The other is cyclo-metaphosphate with *Cmc*2_1_, the phosphoric anions of which have cyclic geometry of [P_4_O_12_]^4−^, and its formula is specially written as KLaP_4_O_12_^26^. In our experiments, we had not found the crystallization of KLaP_4_O_12_. That should be related to the melt composition of the K_2_O−La_2_O_3_−P_2_O_5_ system. According to the experimental result of Belam and Mechergui, KLaP_4_O_12_ crystallizes from the melt with a K:La:P molar ratio of 5:3:58^26^. The melt composition employed by us was apparently different from that in the literature: there were more K_2_O component and less P_2_O_5_ component. This case that the melt composition influences the formation of phase also occurs to BaTeMo_2_O_9_ polymorphous compound reported recently—the *α* and *β* crystal phases can be grown from melts with different compositions^27^.

We adjusted the position of the crucible inside the tubular furnace to ensure that the melt temperature at the bottom had been higher than that at the surface. This issue was important because it assured that it was at the melt surface where the spontaneous nucleation occurred. In our experiment, the melt depth was about 4 cm, and the temperature difference between the bottom and surface of the melt was no less than 10 °C. Owing to surface tension, the crystals usually floated at the melt surface for a rather long time. The largest single crystal of KLa(PO_3_)_4_ grown by us reaches a size of 40 × 35 × 8 mm^3^ and a weight of 18.5 g [Figure 1(a)]. Though a regular shape, it has a poor quality—a lot of inclusions exist inside and it is nearly non-transparent except for the margin. In addition, there are many millimeter-sized crystal growth steps at the other side of the crystal. These results indicate that the crystal grew too fast under the melt cooling rate of 1 °C/1.5 days. When the cooling rate was slowed to 1 °C/2 days, the quality of the crystals was evidently improved, as shown in Figure 1(b). The inclusions reduced, and the transparent areas increased. A wholly transparent and inclusion-free crystal was obtained under the cooling rate of 1 °C/3 days just as presented in Figure 1(c). Consequently, the size of the crystal is smaller due to the slowest growth rate and a short growth time.

In order to choose suitable seed crystal for further growing crystal in the future, it is important to know the orientation of anisotropic KLa(PO_3_)_4_ crystal. Moreover, some key crystal properties are highly dependent on the sample orientation, and the crystals must therefore be exactly orientated when being cut and polished for applications. The crystalline faces of the as-grown crystals in Figure 1 were oriented using an X-ray diffraction goniometer. The measured interfacial angles of the faces were in good agreement with the calculated angles. The morphological schemes of the as-grown crystals were simulated by the WinXMorph software^28^, and were presented in Figure 1 together with the corresponding crystals. The diffraction faces (*hkl*), their inter-planar *d*_(hkl)_, and the observed faces of the as-grown crystals were listed in supplementary information. Crystals (*a*) and (*b*) in Figure 1 have similar morphology. The habit was made up of the crystalline forms {100}, {001}, {110}, {1–10}, {011}, {0–11}, {−101} and {101}. Based on the crystal structure of KLa(PO_3_)_4_, we can find the strongest periodic bond chain (PBC), [001], which influence the growth morphologies of KLa(PO_3_)_4_. The strongest PBC runs nearly parallel with the normal direction of the {001} faces, so these faces have a rapid growth rate. That is why the (001) and (00–1) faces revealed very small and hardly appeared. The {−110} and {1–10} faces of all the crystals were well developed due to a slow growth rate since they are parallel to [001]. Limited by the growth method, the crystal morphologies are not complete, and not each equivalent crystalline face can be found on the crystals. Crystal (*c*) in Figure 1 presents a simple morphology, which is only made of some easily revealed faces, {110}, {1–10} and {101}. That should be related to its very slow cooling rate during crystal growth, and other faces did not have enough time to develop themselves.

### IR and Raman Spectroscopies

**Figure 2:**
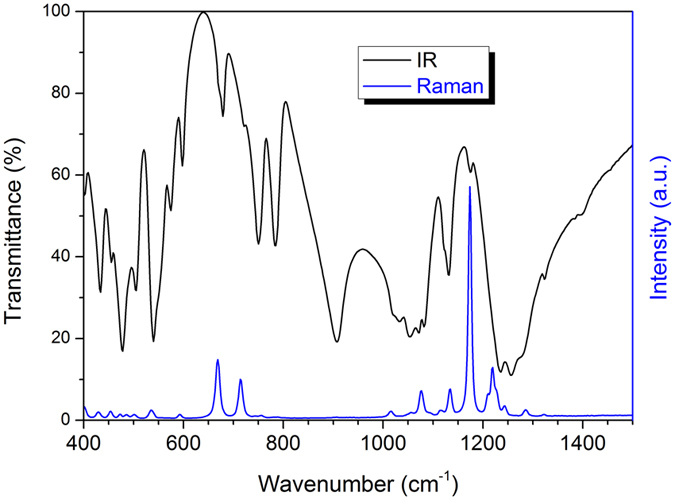
The IR and Raman spectra of KLa(PO_3_)_4_ at room temperature.

Metaphosphates with an O/P ratio of 3 have two types of structural geometry: chain or cycle, whichever is composed by basic structural units of PO_4_ sharing two bridge O atoms. The infrared (IR) and Raman spectra are mainly affected by the geometry and the number of repetitive units (PO_3_)_*x*_ that generate chain or cycle. Different lanthanide or alkali ions in structure only slightly shift the positions of vibration peaks. The IR and Raman spectra of KLa(PO_3_)_4_ crystal at room temperature are shown in Figure 2.

The broad and intense peak around 1255 cm^−1^ in the IR spectrum is assigned to the asymmetrical stretching vibration *ν*_as_ of O−P−O, and the band between 1000 and 1160 cm^−1^ is attributed to the symmetrical stretching vibration *ν*_s_ of O−P−O. We also attribute the sharp and intense peaks at 908 cm^−1^ to the asymmetrical stretching vibration *ν*_as_ of P−O−P. The symmetrical stretching vibration *ν*_s_ of P−O−P is represented by a few peaks between 640 and 800 cm^−1^. When the phosphoric anions of tetra-metaphosphates have cyclic geometry of [P_4_O_12_]^4−^, there is absence of bands in the 750–1000 cm^−1^ region of IR spectrum^29^. Therefore, the strong IR vibration peak around 908 cm^−1^ can indicate that the phosphoric anions of the as-grown crystals have long chain geometry of [PO_3_]_∞_.

The Raman spectrum of KLa(PO_3_)_4_ presents some representative strong peaks around 1174 and 700 cm^−1^. Generally, the strong peak around 1174 cm^−1^ is assigned to the *ν*_s_ of the O−P−O groups, and the weaker peaks around it, which are in the region of 1000‒1300 cm^−1^, are attributed to the symmetrical *ν*_s_ and asymmetrical *ν*_as_ of the same groups. These peaks are ordinary feature to materials constructed from linked PO_4_ tetrahedra. The *ν*_as_ of the P−O−P chain linkage locates around 900 cm^−1^, and the vibrations are so weak that they are hardly observed here. However, the *ν*_s_ (P−O−P) is rather strong and shows two peaks with similar intensities in the region of 650‒750 cm^−1^. They are due to the different positions of lanthanum and potassium ions giving different frequencies of the *ν*_s_ (P−O−P)^30^. For the *ν*_s_ (P−O−P) of cyclotetraphosphate, there is only one strong peak in the same region^31^. As such, the Raman double peaks around 700 cm^−1^ can also be used to identify the structural type of alkali-metal lanthanide metaphosphate.

### Thermal Properties

**Figure 3:**
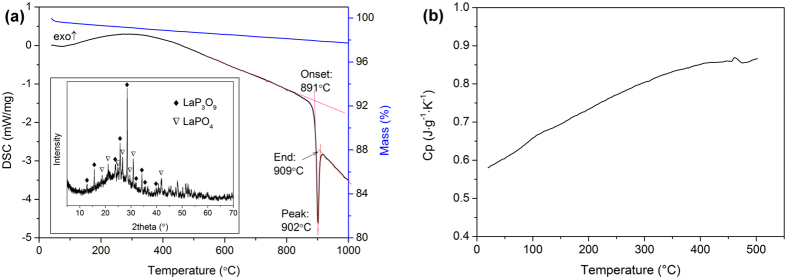
(**a**) The TG-DSC curves of the KLa(PO_3_)_4_ crystal. The inset presents the PXRD pattern of the thermal decomposition products of KLa(PO_3_)_4_. (**b**) The curve of specific heat capacity versus temperature of KLa(PO_3_)_4_.

Ferid^32^ and Jungowska^33^ had investigated the ternary system K_2_O−La_2_O_3_−P_2_O_5_, and both of them conclude that KLa(PO_3_)_4_ melts incongruently. However, they differed on the decomposition temperature (880 °C by Ferid and 840 °C by Jungowska). To specifically determine its decomposition, we pulverized a sample cut from an as-grown crystal and performed a simultaneous thermogravimetry–differential scanning calorimetry (TG-DSC) analysis. As shown in Figure 3(a), the TG-DSC curves of the KLa(PO_3_)_4_ crystal reveal no representative weight loss in the measurement temperature up to 1000 °C, and only a sharp decomposing endothermic peak is observed at 902 °C. That means that KLa(PO_3_)_4_ is very stable before its decomposition, and that there is no polymorphic phase transition between the monoclinic *P*2_1_ and the orthorhombic *Cmc*2_1_. Compared to the literature, our result shows that KLa(PO_3_)_4_ actually has a higher thermal stability. We speculate that the lower decomposition temperatures reported previously could be imputed to the impurity of tested samples then. A thermal decomposing experiment on KLa(PO_3_)_4_ was performed in a Muffle oven. The PXRD study shown in the inset of Figure 3(a) demonstrates that the decomposition products include LaP_3_O_9_, LaPO_4_, and an amorphous phase. The amorphous phase undoubtedly contains phosphorus and potassium oxides because the sample weight remains roughly. So, we deduce that KLa(PO_3_)_4_ decomposes at 902 °C in accordance with the reaction:


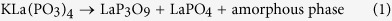


For NLO crystals, specific heat capacity can greatly influence the damage threshold and its further possible applications. Figure 3(b) shows that the constant pressure specific heat capacity of KLa(PO_3_)_4_ varies as a function of temperature. Its specific heat at 25 °C (0.585 J·g^−1^·K^−1^) is higher than that of CsLa(PO_3_)_4_ (0.513 J·g^−1^·K^−1^)^22^. Moreover, the specific heat of KLa(PO_3_)_4_ increases almost linearly from 0.580 to 0.866 J·g^−1^·K^−1^ with temperature increase from 20 to 500 °C, which means that the KLa(PO_3_)_4_ crystal can endure even more thermal energy at high temperature.

### Linear and Nonlinear Optical Properties

**Figure 4:**
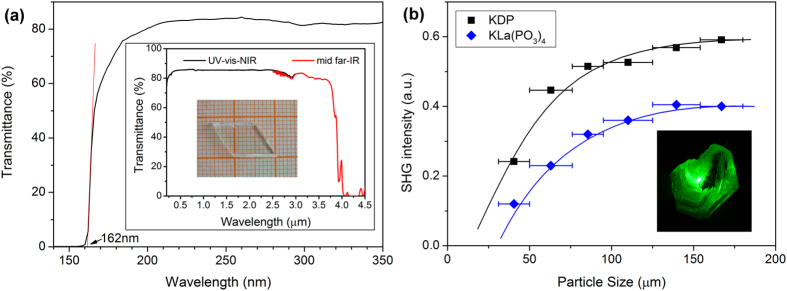
(**a**) The transmittance spectrum of the KLa(PO_3_)_4_ crystal in the VUV region. The inset presents the measured crystal and the transmittance spectra in the UV-vis-NIR and mid far-infrared regions. (**b**) Phase-matching data points with respect to particle size for the KLa(PO_3_)_4_ polycrystalline powder in addition to KDP as a reference at 1064 nm. The drawn curves serve to guide the eye, and do not represent a fit to the data. The inset presents the second-harmonic generation of pulsed Nd:YAG laser radiation on the as-grown KLa(PO_3_)_4_ crystal.

Figure 4(a) presents that the UV cutoff wavelength of the KLa(PO_3_)_4_ crystal is as short as 162 nm. This remarkable deep-UV absorption edge not only is much shorter than those of well-known KTiOPO_4_ and KDP crystals^34^, but also is shorter than those of the phosphate NLO crystals reported recently, such as BaP_3_O_10_Cl (180 nm)^17^, RbBa_2_(PO_3_)_5_ (163 nm)^18^, Ba_5_P_6_O_20_ (167 nm)^19^, and CsLa(PO_3_)_4_ (167 nm)^22^. It is also comparable to those of beryllium borates, such as KBBF (155 nm)^5^, NaSr_3_Be_3_B_3_O_9_F_4_ (170 nm)^9^, and LiNa_5_Be_12_B_12_O_33_ (169 nm)^11^, and those of beryllium-free borates, such as BaAlBO_3_F_2_ (165 nm)^34^ and Ba_4_B_11_O_20_F (<175 nm, from powder diffuse reflectance spectrum)^13^. The short absorption edge constitutes one key feature of this crystal, especially when combined with its acentric structure to generate coherent light below 200 nm if possible. The absorption edge of 162 nm is corresponding to a large band gap of 7.65 eV, which implies that the KLa(PO_3_)_4_ crystal may have a considerable laser damage threshold. The inset of Figure. 4(a) presents the transmittance spectra of the KLa(PO_3_)_4_ crystal in the UV-vis-NIR and mid far-infrared regions. They indicates that the crystal not only is high transparent in the UV-vis-NIR region, but also has a wide optical transparency window of 0.16~4.0 μm. Due to the absorption resonance of the P−O bonds, the cutoff wavelength of the KLa(PO_3_)_4_ crystal in IR region is around 4 μm, which is similar to other alkali metal phosphates^25^.

The fact that KLa(PO_3_)_4_ crystallizes in a acentric space group (*P*2_1_) warrants the study of its NLO property. The powder SHG response of KLa(PO_3_)_4_ was evaluated using a pulse Nd:YAG laser (*λ* = 1064 nm) by the Kurtz-Perry method with KDP as a ref. 35.

Figure 4(b) presents the phase-matching curves of the SHG signal intensity versus particle size for the KLa(PO_3_)_4_ powder samples in addition to KDP as a reference. It can be seen that the SHG efficiency of KLa(PO_3_)_4_ is about 0.7 times that of KDP. This value is comparable to most of the deep-UV NLO phosphate crystals, such as Rb_2_Ba_3_(P_2_O_7_)_2_ (0.3 × KDP), CsLa(PO_3_)_4_ (0.5 × KDP), Ba_3_P_3_O_10_Cl (0.6 × KDP), Ba_5_P_6_O_10_ (0.8 × KDP), and RbBa_2_(PO_3_)_5_ (1.4 × KDP)^17^,^18^,^19^,^22^. Moreover, the results indicate that KLa(PO_3_)_4_ is a type I phase-matchable material in visible region according to the rule proposed by the Kurtz and Perry method^35^. Then the pulsed Nd:YAG laser irradiated an as-grown KLa(PO_3_)_4_ crystal, which was freely rotated for meeting the requirement for phase-matching. When the crystal was only in a particular orientation, the strong SHG green light of the incident Nd:YAG laser radiation was observed by naked eyes [the inset of Figure 4(b)]. That supports the conclusion that KLa(PO_3_)_4_ crystal is phase-matchable in the visible region. It should be noted that the phase-matching behavior in the visible region cannot guarantee its certainty in the violet region due to the refractive index dispersion. A further SHG test at 532 nm, even shorter wavelength, would be necessary for confirm the certainty.

In order to better comprehend the SHG origin of the KLa(PO_3_)_4_ crystal, we calculated the local dipole moments of the KO_8_, LaO_8_, and PO_4_ polyhedra using a bond-valence approach proposed by Poeppelmeier *et al.*^36^,^37^, and the results are summarized in Table 1. Because of the symmetry of the 2_1_ helical axis, the *x* and *z*-components of the dipole moments for all the polyhedra within a unit cell cancel each other out completely, and the *y*-component of the dipole moments constructively adds to a net value of 24.56 Debye. This value is consistent with the moderate SHG behavior in the foregoing SHG test. The dipole moments of PO_4_ polyhedra in KLa(PO_3_)_4_ varies around 4 Debye. It is worth noting that the oxygen polyhedra of alkali metal and rare earth have bigger dipole moments in KLa(PO_3_)_4_, and they are 8.63 and 2.32 Debye, respectively. That is to say, the KO_8_ polyhedron in KLa(PO_3_)_4_ have a much heavier distortion, just as shown in supplementary information. In addition, the *y*-component of the dipole moment of the KO_8_ polyhedron (8.4 Debye) is far bigger than the sum of the *y*-components of all PO_4_ polyhdra (2.43 Debye). Therefore, we speculate that the contribution of KO_8_ to SHG should be much significant. That should be the reason why the SHG efficiency of KLa(PO_3_)_4_ is higher than that of CsLa(PO_3_)_4_, although both of them have infinite [PO_3_]_∞_ chain structures.

### Theoretical Calculation

**Figure 5:**
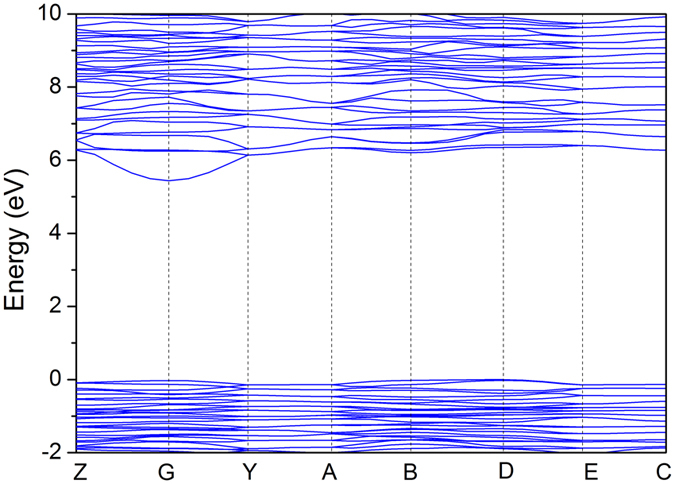
The band structure of KLa(PO_3_)_4_.

To gain further understandings of the electronic structure and optical properties of the KLa(PO_3_)_4_ crystal, the theoretical calculations based on a density functional theory (DFT) method were performed. Figure 5 presented the calculated band structure of KLa(PO_3_)_4_. The tops of valence bands (VBs) and the bottoms of conduction bands (CBs) are located at the D (−0.5, 0.0, 0.5) and G (0.0, 0.0, 0.0) points, respectively. Hence, the KLa(PO_3_)_4_ crystal is an indirect bandgap insulator with a predicted energy band gap of 5.44 eV. The predicted value is smaller than the experimental value (7.65 eV) due to the infamous bandgap underestimation of the generalized gradient approximation (GGA), which does not sufficiently describe the eigenvalues of the electronic states. So, a scissor value of 2.21 eV was applied in the subsequent density of states (DOS) and optical properties calculations.

**Figure 6:**
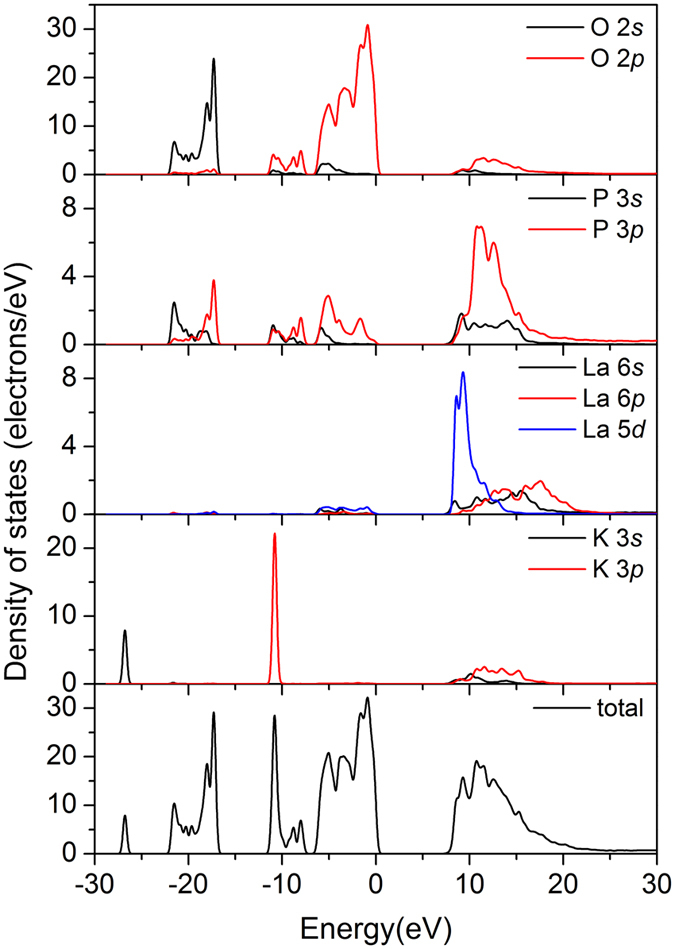
The total and partial densities of states of KLa(PO_3_)_4_.

The total and partial DOS analyses shown in Figure 6 indicate that the O-2*p* states provide the significant contributions to the tops of VBs from 6.9 eV to the Fermi level. The O-2*p* and P-3*p* states produce the VBs from −9.7 eV to −6.9 eV, while the VBs from −12.0 eV to −9.7 eV are mainly composed of the K-3*p* states mixing with a minority of the O-2*p*, P-3*s* and P-3*p* states. Above the Fermi level, the CBs from 5.44 eV to 25.0 eV is chiefly derived from the unoccupied La-5*d* and P-3*p* states, which means that the La and P atoms act primarily as electron donors. Similarly, the O atoms act as electron acceptors because their *p* states are localized below *E*_F_. It is worth noting that the La-5*d* states make more contributions to the bottoms of the CBs than the P-3*p* states. Because the optical response of a crystal mainly originates from the electronic transitions close to the energy band gap, especially between the tops of the VBs and the bottoms of the CBs, it is the La−O groups that mainly determines the magnitude of the bandgap of KLa(PO_3_)_4_. So, we predict that absorption edge of phosphate would be probably blue-shifted if lanthanum component was excluded.

**Figure 7:**
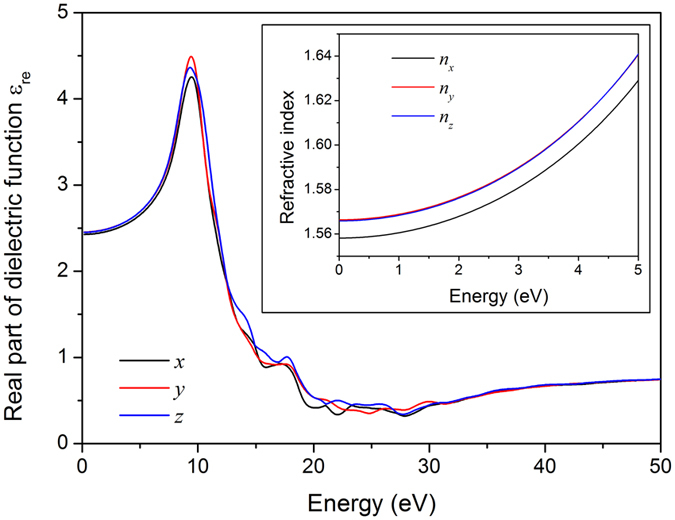
The real part of the dielectric function of KLa(PO_3_)_4_ over the three directions of the principal dielectric axis coordinate system. The inset presents the calculated principal refractive indices.

The calculation and analysis of optical properties for a low-symmetry crystal should be based on the principal dielectric axis coordinate system^38^. For the monoclinic KLa(PO_3_)_4_ crystal, only a single dielectric axis (*y*) is set to the *b* axis. Other two principal dielectric axes (*x* and *z*) are in the *ac* plane; however, they are not associated with any particular crystallographic direction. In accordance with the methodology proposed by Lang and Claus^39^, the directions of the principal dielectric *x* and *z* axes were determined, and they are respectively to be 60.53° anticlockwise and 29.47° clockwise from the crystallographic *c* axis when *ω* equals 0. The optical properties of KLa(PO_3_)_4_, including the complex dielectric function, the refractive indexes, and the second-order susceptibilities, were then calculated in the principal dielectric axis coordinate system. Figure 7 shows the real part of static dielectric function, and the average static dielectric constant *ε*_re_ (0) is 2.445. The refractive indices were calculated as *n*^2^(*ω*) = *ε*_re_(*ω*) and are presented in the inset of Figure 7, which indicates an order of *n*_y_ ≈ *n*_z_ > *n*_x_ in the range of 0‒5 eV. That is to say, the optical indicatrix of the KLa(PO_3_)_4_ crystal more approximates an equiaxial ellipsoid, although it is an optical biaxial crystal. The values of *n*_x_, *n*_y_, and *n*_z_ at 1064 nm (i.e. 1.165 eV) were calculated to be 1.5614, 1.5698 and 1.5693, respectively. Correspondingly, the optical birefringence (Δ*n*) of the KLa(PO_3_)_4_ crystal is 0.0084, which is higher than that of CsLa(PO_3_)_4_ by 24%^22^. That just explains the fact the KLa(PO_3_)_4_ crystal is phase-matchable at 1064 nm, whereas CsLa(PO_3_)_4_ is not.

The space group of the KLa(PO_3_)_4_ crystal belongs to class 2 and has 8 non-vanishing tensors of second-order susceptibility. However, under the restriction of Kleinman’s symmetry, only four independent SHG tensors (*d*_14_, *d*_16_, *d*_22_, and *d*_23_) remain. The values of *d*_14_, *d*_16_, *d*_22_, and *d*_23_ at 1064 nm are 1.62 × 10^−9^, 1.58 × 10^−9^, 1.65 × 10^−9^, and 1.65 × 10^−9^ esu, respectively (*d*_36_ of KDP is 1.1 × 10^−9^ esu). The theoretical results agree with the SHG experimental observation in principle.

## Conclusions

In summary, centimeter-sized crystals of KLa(PO_3_)_4_ with acentric *P*2_1_ space group were successfully grown using the flux method and slow-cooling technique for the first time. The KLa(PO_3_)_4_ crystal is nonhygroscopic and chemical stable. The strong IR vibration peak around 908 cm^−1^ and the Raman double peaks nearby 700 cm^−1^ can be used to distinguish KLa(PO_3_)_4_ from its cyclotetraphosphate isomer. KLa(PO_3_)_4_ is thermal stable till melting incongruently around 902 °C, and the specific heat capacity is 0.585 J·g^−1^·K^−1^ at 25 °C. The SHG efficiency of polycrystalline KLa(PO_3_)_4_ powder is 0.7 time that of KDP. Moreover, it is phase-matchable in the visible region. Structural analysis indicates that the good SHG response is related to the heavily distorted KO_8_ polyhedron apart from the PO_4_ polyhedra. Remarkably, the KLa(PO_3_)_4_ crystal exhibits a deep-UV absorption edge of 162 nm, which is mainly attributed to the electronic transitions between the O and La atoms. To our knowledge, it is the shortest among the UV absorption edges of phase-matchable phosphates. The attributes above make the KLa(PO_3_)_4_ crystal a possible deep-UV NLO material.

## Experimental Section

### Crystal Growth

Due to melting incongruently, the KLa(PO_3_)_4_ crystal cannot be grown from stoichiometric melt. We adopted the flux method and slow-cooling technique to grow KLa(PO_3_)_4_ single crystals. K_2_CO_3_ (AR), La_2_O_3_ (4N), and NH_4_H_2_PO_4_ (AR) with a molar ratio of 3:1:12 were thoroughly mixed in an agate mortar. The mixture was then transferred into a Pt crucible (Φ60 mm × 60 mm), and then was slowly heated to 900 °C to be a melt in a vertical tubular electric furnace. The temperature was held for 2 days to ensure that the melt was homogenized and there were no bubbles. Then the melt was allowed to cool at a rate of 5 °C·h^−1^ until spontaneous crystallization occurred at the melt surface. To reduce the number of crystal nucleus, the melt temperature was oscillated. Subsequently, the melt was cooled at a very slow rate, and the crystal gradually grew near the melt surface. Some days later, the temperature was decreased to room temperature at a rate of 25 °C·h^−1^. Finally, the as-grown crystals were physically separated from the matrix by hot water washing off flux.

### Material Characterization

Microprobe elemental analyses were performed on a field emission scanning electron microscope (FESEM, FEI Helios Nanolab 600i) equipped with an EDS (EDAX TEAM Octane Plus). Crystal density was measured at room temperature (23 °C), using a density unit of Sartorius balance (YDK01−C) by the buoyancy method. PXRD patterns were collected at room temperature by a Fangyuan DX2700 (Dandong, China) powder diffractometer using a graphite monochromatized Cu *K*_α_ radiation in the 2*θ* range of 10°–70° with a step of 0.02°. The crystalline forms that compose the morphological habit were identified and oriented by an XRD goniometer. IR spectra in the range of 4000–400 cm^−1^ were recorded on a Nicolet MagnaIR−560ESP Fourier transform IR (FT-IR) spectrometer employing KBr pressed pellet. Raman spectra were recorded on a Renishaw inVia confocal Raman microscope with an excitation wavelength of 514 nm. TG-DSC analyses were carried out using Netzsch STA 449C in the range of 40–1000 °C with a heating rate of 10 °C·min^−1^. The specific heat capacity was analyzed by DSC using Netzsch DSC 200 F3 between 20 and 500 °C under an N_2_ atmosphere.

Deep-UV optical transmittance spectrum at room temperature was measured on a polished KLa(PO_3_)_4_ crystal at room temperature at the VUV station of Beijing Synchrotron Radiation Facility. The transmittance spectra in the UV–vis–NIR and mid far-infrared regions were recorded using a Cary 2390 spectrophotometer and a Nicolet Magna-IR560ESP FT-IR spectrometer, respectively. SHG testing was performed by using the Kurtz-Perry method^35^. The SHG intensity depended strongly on the particle size of the sample, and thus, polycrystalline KLa(PO_3_)_4_ powder was sieved into a series of distinct size ranges of 31–50, 50–76, 76–95, 95–125, 125–154, and 154–180 μm, respectively. The sample was pressed between glass slides in a 1-mm-thick aluminum cell, and then was irradiated by a pulsed Nd:YAG laser (*λ* = 1064 nm). Sieved KDP with the same particle size ranges was served as a reference material to evaluate the SHG property of KLa(PO_3_)_4_. SHG of the as-grown single crystals were also tested using the same pulsed Nd:YAG laser for verifying whether the KLa(PO_3_)_4_ crystal is phase-matchable.

### Computational Descriptions

The crystallographic structure data of KLa(PO_3_)_4_ reported by Lin *et al.* were used for the theoretical calculations^24^. The band structure, DOS, dielectric function, and optical properties were determined using the total-energy code CASTEP package based on DFT^40^,^41^. The total energy was calculated within the framework of nonlocal gradient-corrected approximations [Perdew-Burke-Ernzerhof (PBE) functional]^42^. The norm-conserving pseudopotential was chosen to describe the interactions between the ionic cores and the electrons^43^. The following valence-electron configurations were considered in the computation: O-2*s*^2^2*p*^4^, P-3*s*^2^3*p*^3^, K-3*s*^2^3*p*^6^4*s*^1^ and La-5*d*^1^6*s*^2^. The cutoff energy for the plane wave basis was set to be 830 eV, and a 3 × 3 × 3 Monkhorst-Pack *k*-point sampling in the Brillouin zone was used for KLa(PO_3_)_4_. The other calculating parameters and convergent criteria were set by the default values of CASTEP code.

The first-order susceptibility can be derived from the dielectric function. The second-order susceptibilities can be derived from the classical anharmonic oscillator (AHO) model^44^,^45^, and are expressed in terms of the first-order susceptibilities as follows:


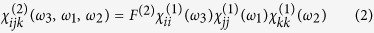


Here, *F*^(2)^ = *ma*/(*N*^2^*e*^3^), where *m* and *e* are the electron mass and charge, respectively, *N* is the number density of atoms in a crystal, and the parameters *a*, which characterizes the nonlinearity of the response, can be obtained from experimental or theoretical estimations.

## Additional Information

**How to cite this article**: Shan, P. *et al.* Crystal growth and optical characteristics of beryllium-free polyphosphate, KLa(PO_3_)_4_, a possible deep-ultraviolet nonlinear optical crystal. *Sci. Rep.* **6**, 25201; doi: 10.1038/srep25201 (2016).

## Supplementary Material

Supplementary Information

## Figures and Tables

**Table 1:** Calculation of the dipole moments of the KO_8_, LaO_8_, and PO_4_ polyhedra in the asymmetric unit of KLa(PO_3_)_4_.

Polar unit	Dipole moment (Debye)			
	*x*-component	*y*-component	*z*-component	Total magnitude
KO_8_	−1.38	8.40	1.40	8.63
LaO_8_	−0.91	1.46	−1.56	2.32
P(1)O_4_	−1.81	−1.86	−0.83	2.73
P(2)O_4_	3.00	3.09	1.04	4.43
P(3)O_4_	3.00	1.65	−1.89	3.91
P(4)O_4_	−3.10	−0.45	−2.13	3.79

Note: The relationship between the Cartesian coordinate system (*xyz*) and the crystallographic system (*abc*) for the KLa(PO_3_)_4_ crystal is *x*//*a*, *y*//*b* and *z*//c^*^.
