# Supplementary material for: Crystal growth and optical characteristics of beryllium-free polyphosphate, KLa(PO3)4, a possible deep-ultraviolet nonlinear optical crystal
Source: Sci Rep. 2016 Apr 29;6:25201. doi: 10.1038/srep25201 (PMC4850429; doi:10.1038/srep25201)
Supplement: Supplementary Information [file srep25201-s1.doc]

**Supplementary Information**

**Crystal growth and optical characteristics of beryllium-free polyphosphate, KLa(PO3)4, a possible deep-ultraviolet nonlinear optical crystal**

Pai Shan^1^, Tongqing Sun^1,4*^, Hong Chen^1^, Hongde Liu^1,4^, Shaolin Chen^1,2,4^, Xuanwen Liu^3^, Yongfa Kong^1,2,4^ & Jingjun Xu^1,2,4^

^1^The MOE Key Laboratory of Weak-Light Nonlinear Photonics and School of Physics, Nankai University, Tianjin 300071, China.

^2^Teda Institute of Applied Physics, Nankai University, Tianjin 300457, China

^3^School of Resources and Materials, Northeastern University at Qinhuangdao, Qinhuangdao 066004, China.

^4^Collaborative Innovation Center of Extreme Optics, Shanxi University, Taiyuan, 030006, China

Correspondence and requests for materials should be addressed to T.S. (email: suntq@nankai.edu.cn)

**Figure S1.** The experimental PXRD pattern of KLa(PO_3_)_4_ observed from pulverized crystal and the standard pattern.

**Figure S2.** The KO_8_ and LaO_8_ polyhedra and 1D [PO_3_]_4n_ zigzag chain along the *c* axis in the unit cell of KLa(PO_3_)_4_. The sky blue arrows indicate the approximate directions and magnitudes of the dipole moments.

**Table S1.** The crystalline forms {*hkl*} observed in the KLa(PO_3_)_4_ crystals and the corresponding *d*_(hkl)_ arranged by decreasing sense.


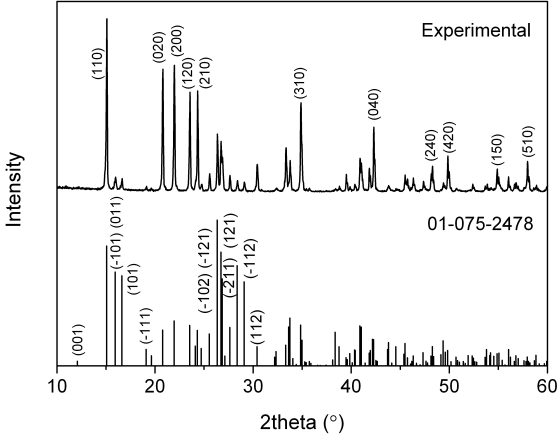


**Figure S1.** The experimental PXRD pattern of KLa(PO_3_)_4_ observed from pulverized crystal and the standard pattern.

**
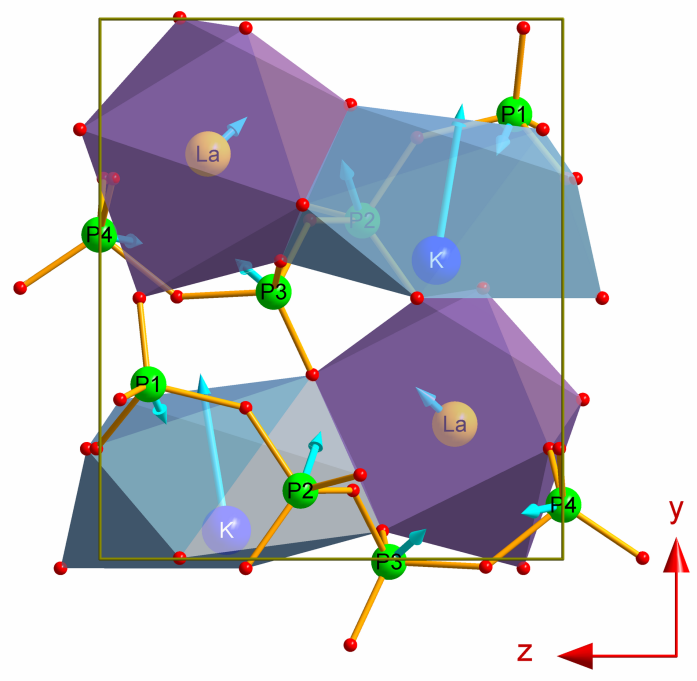
**

**Figure S2.** The KO_8_ and LaO_8_ polyhedra and 1D [PO_3_]_4n_ zigzag chain along the *c* axis in the unit cell of KLa(PO_3_)_4_. The sky blue arrows indicate the approximate directions and magnitudes of the dipole moments.

**Table S1. The crystalline forms {*hkl*} observed in the KLa(PO_3_)_4_ crystals and the corresponding *d*_(hkl)_ arranged by decreasing sense.**

| ***d*_(hkl)_ (Å)** | **Diffraction face  (*hkl*)** | **Crystalline faces {*hkl*}** | **Observed faces on crystals in Figure 1** | | |
| --- | --- | --- | --- | --- | --- |
|  |  |  | **(*a*)** | **(*b*)** | **(*c*)** |
| 8.1001 | (100) | {100} | Y | Y |  |
| 7.3207 | (001) | {001} | Y | Y |  |
| 5.8806 | (110) | {110} | Y | Y | Y |
|  |  | {1-10} | Y | Y | Y |
| 5.5611 | (011) | {011} | Y |  |  |
|  |  | {0-11} | Y | Y |  |
| 5.5370 | (-101) | {-101} | Y | Y |  |
| 5.3313 | (101) | {101} | Y | Y | Y |
| 4.6477 | (-111) | {-111} |  |  |  |
|  |  | {-1-11} | Y |  |  |
